# Preclinical CSF proteomic changes: a milestone in biomarker detection for autosomal dominant Alzheimer’s disease

**DOI:** 10.1038/s41392-024-02109-3

**Published:** 2025-01-20

**Authors:** J. Alexander Ross, Richard Dodel

**Affiliations:** 1 Therapy Research in Neurogeriatrics, Chair of Geriatric Medicine, University of Duisburg-Essen, Essen, Germany; 2 Center for Translational Neuro- and Behavioral Sciences, University Hospital Essen, University of Duisburg-Essen, Essen, Germany

**Keywords:** Neurological disorders, Predictive markers

In a recent study published in *Cell* by Shen et al., changes in the cerebrospinal fluid (CSF) proteome in carriers of known mutations for autosomal dominant inherited Alzheimer’s disease (ADAD) led to the identification of novel, early biomarkers. Based on the changes of six selected proteins a predictive model for disease onset and progression was developed:^1^ SPARC Related Modular Calcium Binding 1 (SMOC1), SPARC Related Modular Calcium Binding 2 (SMOC2), Neuronal Pentraxin 2 (NPTX), Proliferation And Apoptosis Adaptor Protein 15 (PEA15), Glial Fibrillary Acidic Protein (GFAP), and TNF Receptor Superfamily Member 1B (TNFRSF1B).

Recent criteria define Alzheimer’s disease (AD) as a biological process that is first detectable by abnormal biomarkers, which already are measurable when the person is clinically asymptomatic.^2^ The disease progresses and clinical symptoms may appear when sufficient neuropathological damage accumulated. ADAD accounts for approximately 1% of Alzheimer’s disease (AD) cases and is caused by autosomal dominant mutations in the Amyloid Beta Precursor Protein (*APP*), Presenilin 1 (*PSEN1*), or Presenilin 2 (*PSEN2*) genes. ADAD typically manifests earlier than sporadic forms of AD (sAD), with symptom onset often at the ages of 30–50 years. The Dominantly Inherited Alzheimer Network (DIAN) study is a global research initiative focusing on individuals at risk of ADAD. Participants include individuals with known ADAD mutations, as well as their non-mutation-carrying relatives, serving as controls. Many participants are either asymptomatic or in the early symptomatic stages at enrollment, allowing researchers to study disease progression over time. The DIAN and related studies have established that ADAD shares key clinical and neuropathological characteristics with late-onset sAD, including amyloid beta (Aβ) measures, tau, and neurodegeneration. However, due to the genetic determinism of ADAD, pre-symptomatic biomarker studies are feasible, revealing pathological changes decades before cognitive symptoms arise.^3^

An earlier study on ADAD provided a detailed timeline of biomarker changes and cognitive decline, showing that Aβ measures occur first up to 25 years before expected symptom onset. This was followed by changes in cortical metabolism, cognitive decline, and hippocampal atrophy. By tracking ADAD mutation carriers over time, the study established that these biomarker changes unfold sequentially, offering insights into the early disease trajectory and setting a foundation for predicting disease initiation and progression.^4^ Proteomic studies on ADAD have been conducted in the past to map early biomarkers and molecular changes in cerebrospinal fluid (CSF). For instance, a 2023 study used targeted proteomics to detail the temporal evolution of AD pathology, identifying proteins such as SMOC1 and Spondin 1 (SPON1) that increase decades before symptom onset and associating these changes with specific pathological stages.^5^

The study by Shen et al. conducted a comprehensive analysis of CSF and plasma proteomics in individuals with ADAD, leveraging data from the DIAN. The researchers aimed to identify early biomarkers by examining 6163 proteins in CSF and 6022 in plasma, using a cross-sectional approach to simulate longitudinal data. They analyzed samples from 286 mutation carriers (MCs) and 177 non-carriers (NCs), uncovering 137 proteins with significant pseudo-trajectory differences and 227 proteins associated with ADAD mutation status in CSF. In contrast, only three proteins were found to be significantly altered in plasma, while seven were associated with mutation status. The alterations were observable from about seven years in plasma and up to 30 years in CSF before symptom onset. Notably, proteins such as SMOC1 demonstrated changes well before traditional biomarkers like Aβ and tau, with SMOC1 alterations occurring approximately 15 years before established markers. Other notable proteins included NEFL and Neurogranin (NRGN), known markers in Alzheimer’s research, which further validate the study’s results through correlation with established AD pathophysiology.

**Fig. 1:**
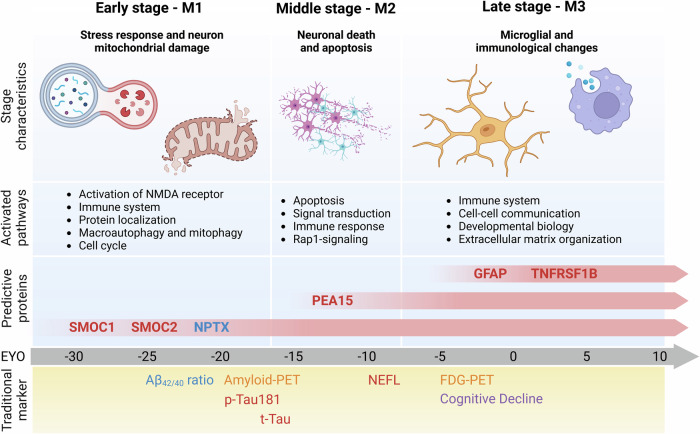
Three early stages of proteomic changes and six selected proteins for a new predictive model in ADAD. Temporal occurrence of characteristic physiological changes during the preclinical phase of ADAD and activation of super-pathways are staged into M1, M2, and M3. For each stage, distinct proteins were selected for the development of a new prediction model, M1: SPARC Related Modular Calcium Binding 1 (SMOC1), SPARC Related Modular Calcium Binding 2 (SMOC2), Neuronal Pentraxin (NPTX); M2: Proliferation And Apoptosis Adaptor Protein 15 (PEA15); M3: Glial Fibrillary Acidic Protein (GFAP), TNF Receptor Superfamily Member 1B (TNFRSF1B). The lower panel indicates the continuum in traditional biomarkers of its defining neuropathological features. Red = elevated expression, blue = decreased expression/ratio, yellow = detection in positron emission tomography, purple = neurological symptoms, EYO = estimated year of onset. This figure was created using BioRender (www.biorender.com; Toronto, Ontario, Canada)

By gene set enrichment analysis these proteins were classified into three distinct modules, representing different stages of disease progression. The M1 module reflected early neuronal damage, while the M2 and M3 modules marked progressive changes linked to neuroinflammation, immune response, and neuronal repair mechanisms. These findings underscore the importance of mitochondrial damage, excitotoxicity, and immune processes as central features of ADAD pathology (Fig. 1). A predictive model was developed based on the six selected proteins (i.e. SMOC1, SMOC2, NPTX, PEA15, GFAP, and TNFRSF1B), yielding a high accuracy (AUC 0.911) in distinguishing MCs from NCs, outperforming conventional biomarkers such as p-Tau181 (AUC:0.755) and Aβ_42_ (AUC:0.709) This model showed potential as a tool for both early diagnosis and monitoring disease progression, demonstrating particular promise as an Aβ-independent diagnostic approach in ADAD. The identification of proteomic changes decades before disease onset represents a significant advancement for early ADAD diagnosis.

While research on ADAD has yielded substantial insights into the molecular and clinical underpinnings of AD, translating these findings directly to sAD presents onsiderable challenges. ADAD and sAD share hallmark neuropathological features, such as Aβ plaques, tau tangles, and progressive neurodegeneration. However, the etiologies and temporal progression of these diseases may differ considerably, leading to limitations in the transferability of ADAD-derived data to sAD. The revealed proteomic and biomarker findings from ADAD research might not appear as early or as uniformly in sAD, where disease initiation is more gradual and less predictable. Therefore, biomarkers identified in ADAD may lack sensitivity or specificity in the broader sAD population. ADAD biomarker candidates such as SMOC1 and GFAP could be tested in sAD patients to evaluate their relevance. Additionally, single-cell sequencing studies could provide insights into whether proteomic pathways highlighted in ADAD are similarly disrupted in sAD. This would bridge the gap between ADAD and sAD, advancing personalized therapeutic strategies.

This study provides a crucial contribution to ADAD research. The identification of proteomic changes up to 30 years before symptom onset emphasizes the importance of an early diagnosis. Targeting the pre-symptomatic stages could provide improved quality of life and significantly delay disease onset and progression. Additionally, the identification of novel biomarkers creates new opportunities for therapeutic development as new therapies aim to reduce Aβ or Tau, their values may falsify the prediction of disease progression. Finally, the use of proteomic pseudo-trajectories provides insight into how molecular changes develop over time. This offers a basis for the prediction of the onset and progression of sAD.

Looking ahead, the future integration of multi-omics approaches, combining proteomics, genomics, transcriptomics, and metabolomics could create a comprehensive overview of the molecular basis and interrelated pathological pathways of ADAD and sAD. From the clinical perspective, the new insights need to be validated in larger cohorts and subsequently implemented in the established diagnostics. Finally, therapeutic exploration should prioritize early interventions targeting mitochondrial dysfunction and synaptic signaling disruptions.

These insights bring us closer to a future where ADAD and sAD are not only understood at their earliest stages but can be prevented before significant neurodegeneration has occurred.
